# The Future of GDNF in Parkinson's Disease

**DOI:** 10.3389/fnagi.2020.593572

**Published:** 2020-12-07

**Authors:** Fredric P. Manfredsson, Nicole K. Polinski, Thyagarajan Subramanian, Nicholas Boulis, Dustin R. Wakeman, Ronald J. Mandel

**Affiliations:** ^1^Department of Neurobiology, Barrow Neurological Institute, Phoenix, AZ, United States; ^2^The Michael J. Fox Foundation for Parkinson's Research, New York, NY, United States; ^3^Department of Neurology and Neural and Behavioral Sciences, Penn State University College of Medicine, Hershey, PA, United States; ^4^Department of Neurosurgery, Emory University, Atlanta, GA, United States; ^5^Virscio, Inc., New Haven, CT, United States; ^6^Department of Psychiatry, Yale School of Medicine, New Haven, CT, United States; ^7^Department of Neuroscience, University of Florida, Gainesville, FL, United States

**Keywords:** Parkinson's disease, gene therapy (GT), neurotrophic factor, neurturin, clinical trial (2.172), GDNF (glial cell line-derived neurotrophic factor)

## Introduction

At the 2019 annual meeting for American Society of Neural Therapy and Repair (ASNTR) a special panel assembled to discuss the future of neurotrophic factor delivery in Parkinson's disease (PD), particularly those factors belonging to the Glial cell line-derived neurotrophic factor family of ligands (GFLs; GDNF and Neurturin). The panel consisted of representatives from academia, industry, and non-profit organizations with primary backgrounds in neurology or neurosurgery and the impetus for the assembly was data from the a recent GDNF clinical trial (Whone A. et al., 2019; Whone A. L. et al., 2019) that utilized an enhanced method of protein infusion to facilitate improved spread of GDNF. Despite preclinical success, this trial, as all previously published trials, failed to demonstrate clinical efficacy (Whone A. L. et al., 2019), leaving the field wondering if there is a future for these neurotrophic factors in PD (Kirkeby and Barker, 2019). This opinion piece will summarize the discussion and the overarching recommendations from the meeting.

## Recent Results From GDNF Trials

Over the last few decades there have been numerous clinical trials utilizing central delivery of GDNF or neurturin via direct protein infusion or overexpression using viral vector-based gene therapy (Merola et al., 2010) with the latest trial reporting dosing of the first patient in September 2020 [Brain Neurotherapy Bio; adeno-associated virus (AAV)-GDNF]. Despite preliminary reports of efficacy in the open-label phase of trials, placebo-controlled studies have failed to replicate any favorable outcomes (e.g., Marks et al., 2008, 2010). Although the reasons behind these apparent failures are unknown, one of the issues may be lack of sufficient target engagement—either via poor diffusion in protein delivery trials (Salvatore et al., 2006) or poor transduction in viral vector trials (Bartus et al., 2011). To that end, the most recent trial rationalized that improving delivery with convection enhanced delivery (CED) might overcome the limitation of insufficient putamenal and nigral drug coverage and achieve improvements in motor function (Whone A. et al., 2019). Regrettably, the results from this trial closely resembled those seen in previous trials—improved ^18^F-DOPA uptake in the absence of clinical improvements. Here, we discuss what additional potential inadequacies have confounded various clinical trials and whether any rational hope remains in regard to utilizing this family of growth factors in the treatment of PD.

## Lost in Translation

The most obvious question that remains is whether neurotrophic factors such as GDNF truly hold disease modifying potential for PD. A wealth of preclinical data supports the notion that GDNF may prevent or retard nigrostriatal degeneration. Moreover, preclinical and human clinical data clearly indicate that GFLs are, in fact, CNS dopaminergic trophic factors. Therefore, treatment with GFLs should promote survival of striatal dopamine (DA) innervation and thereby improve the motor symptoms of PD. So why have clinical trials utilizing this approach largely failed?

One key aspect of GDNF and similar factors is that the therapeutic mode of action is not fully defined, and that the degenerating PD brain may be resistant to the neuroprotective potential of these proteins. The lack of clarity on GDNF's mechanism of action may be causing issues in appropriate model selection for preclinical therapeutic testing. For instance, GDNF preclinical data is largely based on acute, toxin-induced models–such as 6-hydroxydopamine and MPTP. While administration of GDNF under these settings have provided both neuroprotection and neurorestoration, the same claims of GDNF efficacy have not been substantiated in other models of PD—such as in alpha-synuclein (α-syn) overexpression models (Decressac et al., 2011). In fact, work in the AAV α-syn overexpression model shows that GDNF exhibits no neuroprotective effect (Decressac et al., 2011).

There is some controversy as to the cause of this resistance to neuroprotection. On one hand, the lack of GDNF-induced beneficial effects in the α-syn model has been argued to be due to the downregulation of Nurr1 and its downstream product, the GDNF receptor component receptor tyrosine kinase (RET) (Decressac et al., 2012), although other groups have failed to reproduce the downregulation of Nurr1. Importantly, some key caveats to α-syn overexpression models lie in the finding that α-syn mRNA is in fact decreased in PD and few have reported changes in Nurr1 and RET in human disease (Chu et al., 2006; Su et al., 2017). Moreover, this and other α-syn models of PD overexpress α-syn by 4–10 times normally seen in human studies. Although animal models provide valuable insight into certain disease processes, it is clear that the PD field suffers from a lack of clinically-predictive animal models that faithfully recapitulate all key aspects of parkinsonian neurodegeneration and disease progression. Thus, until we have models more encompassing of the etiopathological features of PD, future reports of preclinical efficacy, or the lack thereof, must be interpreted with caution.

In regard to translation, it is also important to note that the chief risk-factor in PD is age (Collier et al., 2011), yet a majority of preclinical studies have largely neglected this crucial variable. However, it is clear that age alters the local environment, and confers impediments in delivery modalities such as viral vector transduction (Polinski et al., 2016), amongst others. In addition, many intracellular processes change with aging. Thus, performing the preclinical experimentation in models whose age corresponds to that of the average human patient is as important as choosing the model with the most appropriate pathological insult. Nonetheless, it is important to note that GDNF retains at least some function with advanced age as intraputamenal infusion into aged monkeys reduces age-related motoric deficits (Maswood et al., 2002).

Another equally important variable is disease duration. It is well-known that PD patients with longer duration of disease have more disease related complications, and such patients are the target for surgical experimental therapeutics for valid reasons of clinical morbidity. However, this variable introduces a critical barrier to translating animal studies to humans as animal studies do not recapitulate the longevity of disease duration as they are cost prohibitive. Also, in the open label studies as well as in the blinded placebo controlled studies, the average age of onset of disease was much younger than the average age (<50) of onset of PD (Gill et al., 2003; Slevin et al., 2005; Lang et al., 2006). This younger age of onset for PD represents a unique subpopulation as has been recognized by many contemporary researchers (Mehanna and Jankovic, 2019; Espay et al., 2020). This raises the important question if such younger onset patients are the best candidates and if they are indeed chosen based on preclinical age equivalency, then, the GDNF intervention must be performed much earlier in the course of their illness (the mean duration of illness was 10 years in these studies). This raises important ethical issues of risk vs. benefits in early onset PD subjects from invasive neurosurgical interventions. Early onset PD patients have a slower disease progression trajectory and so are treated effectively with pharmacotherapy during this “honeymoon” period that lasts well over 5 years. Yet, based on preclinical testing data, treating these patients earlier within 5 years of disease onset may be the best possible use of GDNF. The only possible ethical solution to this conundrum is to reduce the risks of the neurosurgical intervention. Developing less invasive and more safe methods for intracranial delivery of either GDNF protein or GDNF delivery vectors will allow testing such therapies in early disease in such younger patients with ethical equipoise.

## Have We Performed the Right Clinical Trial?

One question in the neurotrophic factor field has always been that of the timing of neurotrophic factor administration. Studies that utilized toxin-induced models clearly demonstrated that GDNF must be administered prior to, or during the insult, in order to achieve efficacy. Administration later may enhance the dopaminergic tone of nigral neurons, but does not provide neuroprotection (Mandel et al., 1997, 1999; Salvatore et al., 2004; Manfredsson et al., 2009a). There is clear evidence from human trials that GFLs can induce DA dendritic sprouting (Love et al., 2005; Kordower et al., 2013) or F-dopa uptake (Gill et al., 2003; Whone A. L. et al., 2019). Therefore, if the theorem that increasing striatal DA should confer therapeutic benefit is correct, then it may be that GFLs are biologically effective but have not reached a necessary threshold of striatal DA regeneration to achieve this benefit. Intervention at earlier stages of disease when more of the nigrostriatal DA pathway is intact or has not undergone plastic changes due to ongoing degeneration, should give GFLs a greater opportunity to reach this threshold of striatal regeneration to provide clinical benefit.

Nevertheless, during the early days of neurotrophic factor delivery, questions regarding the integrity of the nigrostriatal system during disease progression remained. It was not until recently that histopathological characterization by Kordower et al. clearly delineated the significant degree of nigrostriatal denervation during the years immediately following diagnosis (Kordower et al., 2013). Thus, the GFL clinical trials to date targeting late stage patients in Hoehn & Yahr stage 3–4 (where reports have been available) clinical trials are seemingly at odds with the preclinical studies administering the intervention when the nigrostriatal system is mostly intact. To that fact, how many times have we heard variations on the statement “you cannot save what is no longer there”? Thus, the only way to reconcile the field is to test the neuroprotective potential of GDNF and neurturin in early stage patients. During our discussion, it was proposed that the quintessential clinical trial would be performed in patients with unilateral onset, prior to contralateral progression. This, of course, yet again brings up the question about patient safety and advocacy.

## Safety of GFLs

Following diagnosis, PD progresses slowly on average, and pharmacological restoration of the dopaminergic tone in the caudate/putamen (e.g., with Sinemet) provides a fairly lengthy “honeymoon period.” Thus, performing a rather complex neurosurgical procedure at a time shortly following diagnosis is not to be taken lightly. This provides an ethical dilemma whereby a clinician is faced with a patient that will maintain acceptable quality of life for some time, yet the disease will progress relentlessly albeit asymmetrically. At this point, how can you justify the testing of an invasive therapeutic paradigm that remains unproven in PD? Neurosurgical experience and advances would support lowering intervention thresholds. Safety data from a wealth of procedures with an indwelling lead/cannula–such as deep brain stimulation (DBS) where a number of anatomical locations, including deep structures, have been targeted—support the lower thresholds as a relatively low rate of serious adverse events are now reported (Budman et al., 2018). In line with DBS safety, striatal intraparenchymal fetal mesencephalic transplantation has demonstrated that the neurosurgical procedure itself is very safe (Lindvall, 2015). Still, despite all the current improvements with stereotactic neurosurgical techniques, one can argue that the risks of a neurosurgical intervention do not match up with the risks associated with currently effective pharmacotherapy in early stages of PD. Therefore, the justification to perform such a procedure must provide disproportionately high benefit to the risks or the risks themselves must get mitigated via the use of less invasive methods of delivery.

Moreover, even if currently optimized surgical methods are used with the least possible adverse events, there are still open questions as to the safety of GDNF itself. Although all indications from preclinical research into neurotrophic factors belonging to this family of proteins suggest that GDNF is safe, perhaps the most compelling data comes from the long-term safety profile of Neurturin, GDNF and other gene therapy-based candidates in human clinical trials (Tenenbaum and Humbert-Claude, 2017; Chu et al., 2020). Nevertheless, there could be consequences of long-term activity with the possibility that secondary issues such as aberrant sprouting of neurons (Georgievska et al., 2002) may lead to a new set of symptoms. Certainly, the use of a clinical approach that allows for cessation of protein delivery (i.e., cannulation/pump infusion, or regulated vectors) would provide a safety mechanism whereby treatment could be halted in the case of an adverse event.

## Disease Diagnosis and Tracking of Progression

Most panelists agreed that GFL delivery could be clinically therapeutic if treatment were initiated earlier in disease progression for PD patients. However, even if all clinicians would agree that the delivery of GFLs was of a similar risk to pharmacological treatment (an agreement that is not in place at present), it is currently impossible to reliably detect very early PD (Rizzo et al., 2016). Despite being easily recognized in the public eye, PD is a rather complex disorder, and early diagnosis is not unequivocal (Berg et al., 2018). In fact, a diagnosis of early PD is extremely uncertain, especially when performed outside of a specialty movement disorders Center of Excellence. For instance, other neurodegenerative disorders such as multiple system atrophy (Krismer and Wenning, 2017) and progressive supranuclear palsy (Owolabi, 2013) can often times be misdiagnosed as PD early in the course of disease (Tolosa et al., 2006). This is obviously a complication that makes clinical trial design for early PD increasingly complex. Along the same lines, disease modifying clinical trials in PD, especially early in the disease, are hampered by the fact that there are no good metrics whereby one can measure progression without very large sample sizes or utilizing exceptionally long trial periods. Moreover, trials thus far have been powered to detect improvement in the Unified Parkinson Disease Rating Scale (UPDRS) when perhaps we should be looking for stabilization in decline. Finally, PD is also an extremely heterogenous disorder: Progression rates vary widely, there is heterogeneity in the predominant symptom (e.g., tremor-dominant vs. gait/balance-dominant) that may not respond the same to GDNF, or may not be homogeneous enough for current progression markers (like UPDRS), to detect changes, Thus, as crucial as future biomarkers are in PD for earlier definitive diagnosis (Parnetti et al., 2019) and to track progression, they will be equally important to enable definitive clinical trials in early disease. Such a shift in treatment paradigms would have the greatest impact for PD in the immediate future.

## Alternatives to GFLs

Although GDNF and neurturin undoubtedly has undergone the highest scrutiny of all potential trophic factors in PD, there are alternatives worth mentioning. Damage to the striatum results in increased astrocytic production of ciliary neurotrophic factor (CNTF), which belongs to the interleukin-6 family of neuropoietic cytokines. CNTF signaling occurs via a variety of heteroreceptors complexes following binding to CNTF receptor alpha (CNTFRα) (Schuster et al., 2003). Although the exact signaling mechanism is unknown, increased CNTF can protect DA neurons from toxicity, both via direct interaction with neurons as well as by reducing the inflammatory potential of microglia (Hagg and Varon, 1993; Nam et al., 2015; Baek et al., 2018). The closely related trophic factors mesencephalic astrocyte-derived neurotrophic factor (MANF) (Petrova et al., 2003) and cerebral/conserved dopamine neurotrophic factor (CDNF) (Lindholm et al., 2007) similarly provide neuroprotection in various animal models of PD (Airavaara et al., 2012). The exact mode of action of these proteins is unknown, although neuroprotection seems to be, at least in part, conferred via modulation of endoplasmic reticulum stress and autophagy (Zhang et al., 2018). A clinical trial is currently ongoing (ClinicalTrials.gov Identifier: NCT03775538) assessing the safety of putamenal delivery of CDNF (Huttunen and Saarma, 2019) and anecdotal reports suggests that the treatment has been well-tolerated. Finally, small molecule GDNF family receptor (GFR) agonists are being investigated as a potential alternative to the invasive neurosurgical approach otherwise required (Ivanova et al., 2018). However, GFR receptors are heavily expressed in organs throughout the body [The Human Protein Atlas (Uhlen et al., 2015)]. For example, an intracerebroventricularly delivered GDNF trial was halted due to side-effects (Nutt et al., 2003) which is likely due to GDNF's actions in hypothalamus (Manfredsson et al., 2009b). Thus, GDNF administration for PD likely requires site-specific putamenal delivery rendering this strategy the rare case where intraparenchymal delivery is more advantageous than a global small molecule paradigm. Nonetheless, regardless of the therapeutic modality one chooses, the same critical GFL safety factors discussed above apply.

## Conclusions

In summary, the panel participants, as well as the audience, expressed cautious optimism for the future of neurotrophic factors, maintaining that GDNF remains a highly promising target in the treatment of PD progression. The preclinical data remain strong, and we simply may not have unleashed the full potential of these proteins, because they have thus not been properly delivered and tested in the context of human disease at feasible points of intervention. Surely, recent improvements such as enhanced vector biodistribution (Kanaan et al., 2017; Davidsson et al., 2019) and less invasive delivery techniques such as focused ultrasound-assisted delivery (Noroozian et al., 2019), are moving us closer to the reinvention of clinical trials. Nevertheless, the path forward is not clear cut, and with current means at our disposal, the execution of early stage clinical trials may not be feasible. It is very possible that the repeated failure to find positive GFL-based clinical trial outcomes mar the field and effectively prohibit future trials from being proposed due lack of financial interests and/or negative public perception. What will the threshold be for investing in new and redesigned trials that are likely to be more expensive than those in the past? In essence, the future of GFL treatment to intervene in the progression of PD symptoms is dependent on significant improvements to preclinical models, improvements to clinical striatal delivery methods, discovery of alternate less invasive methods, improvements to very early PD diagnosis, and especially improvements to PD clinical trial design that would facilitate the prosecution of conclusive clinical trials.

## Author Contributions

FM and RM wrote the first version of this manuscript. All authors contributed to the editing of subsequent drafts.

## Conflict of Interest

DW was employed by Virscio, Inc. The remaining authors declare that the research was conducted in the absence of any commercial or financial relationships that could be construed as a potential conflict of interest.
